# Butyrate induces higher host transcriptional changes to inhibit porcine epidemic diarrhea virus strain CV777 infection in porcine intestine epithelial cells

**DOI:** 10.1186/s12985-024-02428-5

**Published:** 2024-07-11

**Authors:** Zhen Zhong, Yaqin Zhang, Xuting Zhao, Chunbao Zhou, Shubin Zhu, Jiayun Wu

**Affiliations:** Jiangsu Agri-Animal Husbandry Vocational College, Taizhou, 22530 China

**Keywords:** Pigs, Porcine epidemic diarrhea virus, Butyrate, RNA-seq

## Abstract

Newborn piglets’ health is seriously threatened by the porcine epidemic diarrhea virus (PEDV), which also has a significant effect on the pig industry. The gut microbiota produces butyrate, an abundant metabolite that modulates intestinal function through many methods to improve immunological and intestinal barrier function. The objective of this investigation was to ascertain how elevated butyrate concentrations impacted the host transcriptional profile of PEDV CV777 strain infection. Our findings showed that higher concentrations of butyrate have a stronger inhibitory effect on PEDV CV777 strain infection. According to RNA-seq data, higher concentrations of butyrate induced more significant transcriptional changes in IPEC-J2 cells, and signaling pathways such as PI3K-AKT may play a role in the inhibition of PEDV CV777 strain by high concentrations of butyrate. Ultimately, we offer a theoretical and experimental framework for future research and development of novel approaches to harness butyrate’s antiviral infection properties.

## Introduction

Porcine epidemic diarrhea (PED) is an acute, highly contagious and contact intestinal disease caused by porcine epidemic diarrhea virus (PEDV) that affects pigs of all ages and is mainly characterized by vomiting, watery diarrhea and dehydration in infected pigs. Piglets are highly morbidly and fatally affected by PEDV infection, which primarily affects small intestine epithelial cells in vivo. Most of the infected piglets die within 7 days after birth, and the mortality rate of young piglets was as high as 80% [1]. Since 2010 and 2013, PED outbreaks in East Asia and North America have rekindled interest in the 1978-discovered swine coronaviruses [2]. PEDV belongs to the genus *Alphacoronavirus* of the Coronavirus family, which is approximately 28 kb in length (excluding the poly A-tail) that encodes four structural proteins and nonstructural proteins [3]. One of the main methods to prevent PEDV infection in pigs since the PED outbreak has been vaccination. However, since 2010, the emergence of highly mutated PEDV strains has seriously hampered the effectiveness of vaccination, and the use of vaccines has not been able to lower the incidence and lethality of PEDV in piglets [4]. The search for new anti-PEDV strategies is now crucial to the pig industry’s continued growth.

Numerous studies and clinical observations have demonstrated the importance of the gut microbiota in the pathophysiology of acute respiratory distress syndrome and sepsis, and the development of numerous diseases is linked to the decrease of gut bacterial diversity that results in ecological dysregulation [5, 6]. Functionally, these alterations in the microbiota’s makeup have been linked to a reduction in the synthesis of short-chain fatty acids (SCFAs). It has been shown that the number of microorganisms involved in SCFAs production, including *E. faecalis*, *Clostridium perfringens* and *Fusobacterium cholerae*, is reduced in fecal samples from COVID-19 patients compared to healthy controls. Therefore, there is proof that alterations in the microbiota, such as a decrease in SCFA-producing bacteria, are linked to the existence and/or infection of SARS-CoV-2 in the gut [7]. Studies have not been conducted to determine whether these modifications’ impact on SCFAs is connected to infection.

The gut microbiota constitutes the assemblage of microorganisms residing in the gastrointestinal tract of animals, and it plays a crucial role in maintaining the overall well-being of the host organism. SCFAs are produced through the process of fermenting dietary fiber. They are saturated fatty acids that have a carbon chain length ranging from one to six atoms [8]. SCFAs play a crucial role in facilitating the interaction between the host and the microbiota. Previous research has documented the capacity of these molecules to modulate the synthesis of antimicrobial peptides and mucus, as well as influence intestinal permeability and activate the mucosal immune system [9]. Furthermore, it is important to note that SCFAs have a positive impact on gastrointestinal health. Additionally, SCFAs that are produced in the intestine are easily absorbed into the bloodstream and play a crucial role in regulating various organ functions. These functions include energy utilization and metabolism, inflammation, as well as the regulation of the gut-brain axis [10, 11].

A previous study has demonstrated that low concentrations of SCFAs ranging from 10 µM to 1 mM can effectively inhibit the replication of PEDV [12]. However, this study did not investigate the functional mechanism of high concentrations of SCFAs, and the roles and mechanisms of high-concentration SCFAs in porcine coronaviruses, particularly in PEDV, remain unclear. Therefore, the present study aimed to examine the impact of various high concentrations of SCFAs on the replication of PEDV. Specifically, the study focused on investigating the effects of elevated levels of butyrate on the transcriptional regulation of porcine small intestinal epithelial (IPEC-J2) cells during PEDV infection. Furthermore, we have placed significant emphasis on the emerging role of gut-derived metabolites in the infection process of PEDV, with the aim of providing valuable recommendations for future breeding efforts focused on disease resistance.

## Materials and methods

### Cell lines, virus, and plasmids

IPEC-J2 cells were generously provided by Prof. Wenbin Bao from Yangzhou University. IPEC-J2 cells were grown in DMEM (Thermo Fisher Scientific, Waltham, MA) supplemented with 10% FBS (GIBCO) and 1% penicillin/streptomycin (Invitrogen, Waltham, MA) at 37 ℃ with 5% CO_2_. IPEC-J2 cells underwent pretreatment with SFCAs, specifically 10 mM acetate, 10 mM propionate, and 5 mM butyrate, for a duration of 24 h before being infected with the virus. The PEDV CV777 strain was maintained by our laboratory and cultured in Vero cells following the previously described protocol [13]. Briefly, when Vero cells were cultured to a density of about 90%, PEDV was added and incubated for 2 h, the virus supernatant was then removed, and the culture medium was replenished. After 72 h, the virus solution was frozen and thawed three times, centrifuged, and filtered with a 0.45 μm filter. Regarding the PEDV infection in IPEC-J2 cells, the cells were subjected to PEDV infection at a multiplicity of infection (MOI) of 0.1. The PEDV inoculum was removed at 2 h post-infection (hpi), and the cells were subsequently collected at 24 hpi for further experimentation.

### Cell viability assay

IPEC-J2 cells were seeded into 96-well plates at a density of 3 × 10^3 cells/well. They were treated with SCFAs at concentrations of 0, 0.5, 1, 2, 5, 10, and 20 mM. The cells were then analyzed using a Cell Counting Kit-8 (Beyond Biotechnology) 24 h after inoculation. To each well, 10 µL of CCK-8 solution was added and incubated in an incubator for 2 h. Absorption was detected at 450 nm using a microplate reader.

### Real time-quantitative PCR

The extraction of total RNA of IPEC-J2 cells was conducted using the Trizol method. Complementary DNA (cDNA) synthesis was performed using the PrimeScript RT reagent kit (TaKaRa). Real-time quantitative PCR (RT-qPCR) was performed using the qPCR SYBR Green Master Mix (Vazyme). The PEDV *M* gene primer was designed using the Primer Premier 5.0 software (Premier Biosoft, Palo Alto, CA, USA). The forward primer sequence is 5’-TCCCGTTGATGAGGTGAT-3’, and the reverse primer sequence is 5’-AAGCATTGACTGAACGACC-3’. GAPDH was employed as the reference gene for RT-qPCR. The forward primer sequence used was 5’-ACATCATCCCTGCTTCTACTGG-3’, and the reverse primer sequence employed was 5’-CTCGGACGCCTGCTTCAC-3’. The relative quantification results were determined utilizing the comparative Ct (2-ΔΔCt) method.

### Western blot assay

IPEC-J2 cell proteins were extracted and quantified using the BCA protein assay kit (Beyotime Biotechnology). A total of 20 µg of protein was separated on 10% SDS-PAGE gels and subsequently transferred to 0.22 μm PVDF membranes (Millipore). The membranes were blocked with 5% skim milk powder and subsequently incubated with PEDV N antibodies (Medgene Labs) and GAPDH antibodies (Proteintech) at a temperature of 4 °C overnight. The membranes were subsequently incubated with the appropriate secondary antibodies, and an enhanced chemiluminescence (ECL) detection system from Bio-Rad was employed to visualize the protein bands.

### TCID_50_ assay to detect titration of PEDV

The culture supernatants of the PEDV-infected cells under different SCFAs treatments were collected after 24 h of infection and serially diluted at a 10-fold (10^(-1) ~ 10^(-7) ) dilution. Vero cells were seeded into 96-well plates and added with 100 µL of serial dilutions of PEDV. PEDV-infected cells were cultured for 6 days, and the cytopathic effect was observed daily. Viral titers were quantified using the Reed–Muench method, which measures the 50% tissue culture infectious dose (TCID_50_).

### Data analysis of RNA-seq

IPEC-J2 cells were seeded in 6-well plates, after reaching 90–100% confluence, 5 mM butyrate was pretreated for 24 h. The control group was left untreated. Subsequently, the cells were infected with PEDV using a MOI of 0.1 for 24 h. The cells were then collected and divided into 4 groups: butyrate treatment + PEDV infection, butyrate treatment only, PEDV infection only, and negative control (NC), with 3 replicates in each group. RNA isolation of IPEC-J2 cells was performed followed by the Trizol method. Duplicate samples were obtained and utilized to construct the library using the NEBNext^®^ ultraTM RNA library prep kit for Illumina^®^ (NEB). The AMPure XP system (Beckman Coulter) was employed for the purification of fragments with a length ranging from 250 to 300 bp. The PCR process was subsequently conducted. The Agilent Bioanalyzer 2100 system (Agilent Technologies), was employed for the purification of the PCR products. RNA-seq analysis was conducted using an Illumina Hiseq platform following established protocols. The threshold for statistical significance was set as a *P*-adjust value < 0.05.

### Enrichment analysis

Data normalization was carried out by LOWESS fitting on an M versus A plot [14]. KEGG analysis was performed to explore the pathways enriched with differentially expressed genes (DEGs). The DEGs were analyzed using the Kyoto Encyclopedia of Genes and Genomes (KEGG) database to identify all the pathway terms involved. The significance level of each pathway term was calculated using the Fisher test, and pathway terms with a *P*-adjust value < 0.05 were considered statistically significant.

### Statistical analysis

Data were analyzed by SPSS software for statistical analysis. A two-sided Student’s t-test was used to analyze the differences between the two groups, and standard analysis of variance (ANOVA) was used for more than three groups. In all analyses, *P < 0.05; **P < 0.01; ***P < 0.001 for the comparison of the indicated treatments.

## Results and analysis

### SCFAs promote PEDV CV777 strain replication in IPEC-J2 cells

**Fig. 1:**
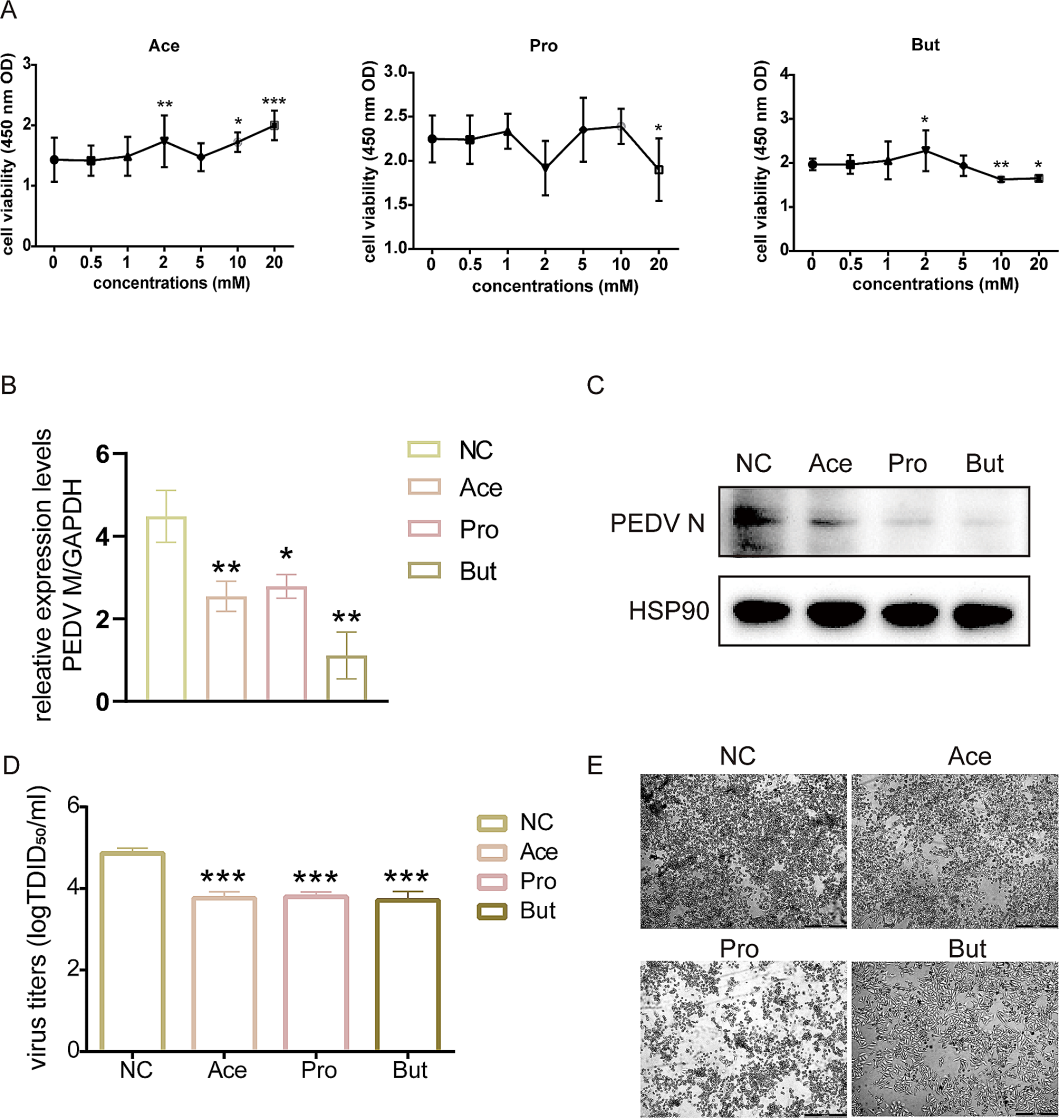
Illustrates the promotion of PEDV replication in IPEC-J2 cells by SCFAs. (**A)** Effect of SCFAs on IPEC-J2 cell viability detected by CCK-8 assay, *n* = 6. (**B**) The impact of various SCFAs on the expression of the PEDV *M* Gene. (**C**) The impact of various SCFAs on PEDV N protein expression. (**D**) TCID_50_ assay was used to determine PEDV virus titers among SCFAs treatments and negative control groups. Viral titers were quantified by a TCID_50_ using the Reed–Muench method. (**E**) IPEC-J2 cell lesion diagram. Light microscopy was used to observe lesions in SCFAs-pretreated and PEDV-only infected IPEC-J2 cells 24 h after PEDV infection. The rounded, crumpled cells are the diseased cells. Experiments for B-C were first performed by pre-treating IPEC-J2 cells with 10 mM acetate, 10 mM propionate, and 5 mM butyrate for 24 h followed by 24 h of PEDV infection. NC: negative control, Ace: Acetate, Pro: Propionate, But: Butyrate. *P < 0.01; **P < 0.001

Previous research has demonstrated that low levels of SCFAs can effectively hinder the infection of PEDV in IPEC-J2 cells. However, the impact of high concentrations of SCFAs on PEDV infection has not been explored yet. Therefore, this study aimed to further investigate the impact of elevated levels of SCFAs on PEDV CV777 strain infection. We initially investigated the impact of SCFAs on IPEC-J2 cell activity. The CCK-8 assay revealed that acetic acid did not inhibit IPEC-J2 cell activity at 20 mM, while propionic acid significantly inhibited IPEC-J2 activity at 20 mM (*P* < 0.05). Moreover, butyric acid exhibited a significant inhibitory effect on IPEC-J2 cells even at 10 mM (*P* < 0.01) (Fig. 1A). Therefore, 10 mM acetate, 10 mM propionate, and 5 mM butyrate were selected for pretreating IPEC-J2 cells for 24 h before infection with PEDV at MOI of 0.1 in this study. The findings indicated that elevated levels of SCFAs significantly inhibited the expression of the PEDV *M* gene (*P* < 0.05) (Fig. 1B). Additionally, the western blot analysis demonstrated a notable inhibition in the expression of the PEDV N protein (Fig. 1C). The TCID_50_ results indicated that all three SCFAs significantly reduced the viral titer of PEDV (*P* < 0.001). Light microscopic observations showed that the lesions were most prominent in IPEC-J2 cells that were not treated with SCFAs. Among the SCFAs examined, it was found that butyrate exhibited the most pronounced impact.

### Butyrate affects the transcription of IPEC-J2 cells more than PEDV CV777 strain infection

**Fig. 2:**
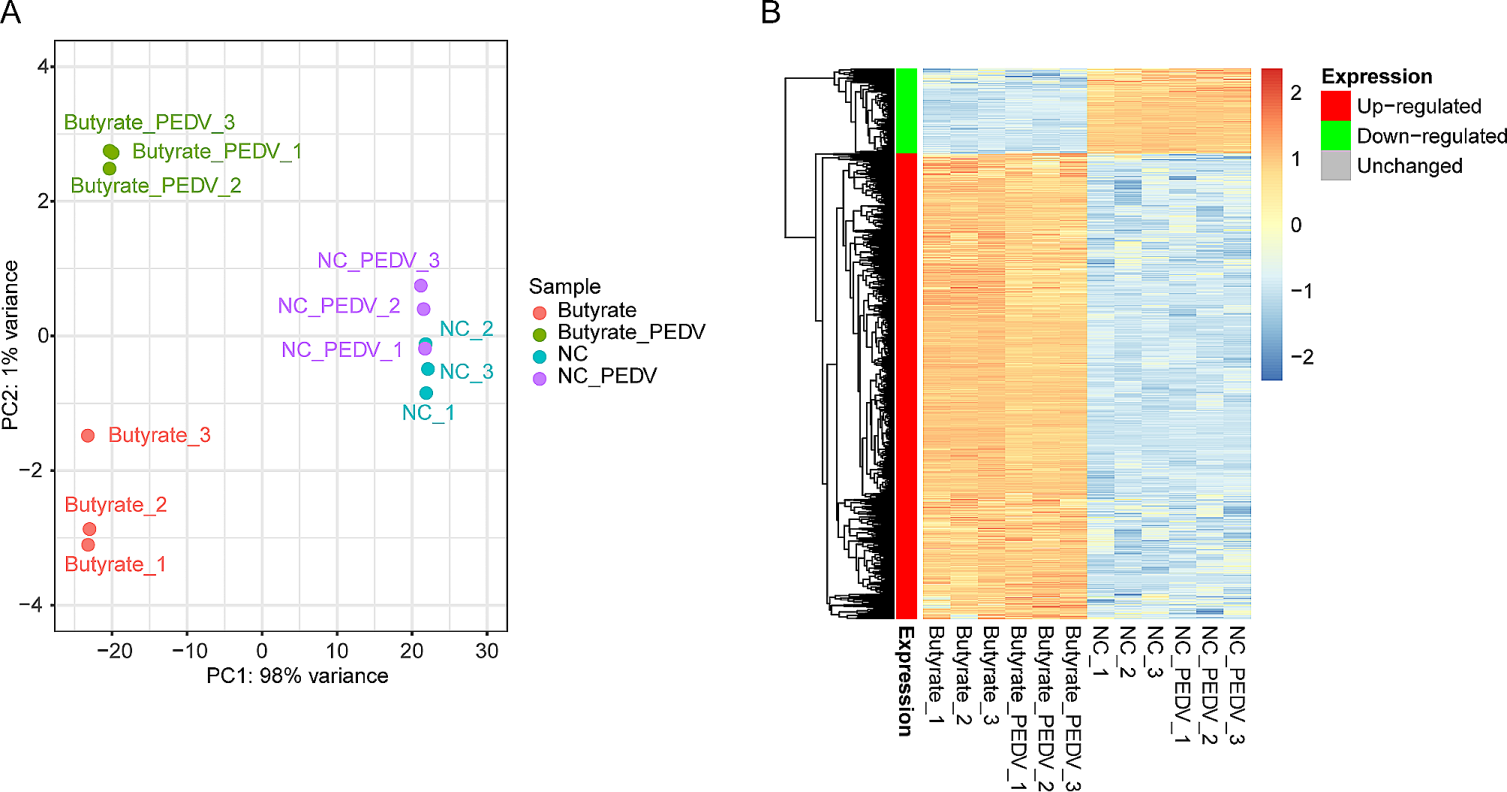
Overall transcriptional alterations in IPEC-J2 cells induced by the administration of butyrate during the process of PEDV infection. (**A**) PCA analysis of all RNA-seq samples. (**B**) Heatmap of gene expression across various groups. For RNA sequencing, IPEC-J2 cells were pretreated by 5 mM butyrate for 24 h, the control group was left untreated. Subsequently, the cells were infected with PEDV using a MOI of 0.1 for 24 h. The cells were then collected and divided into 4 groups: butyrate treatment + PEDV infection, butyrate treatment only, PEDV infection only, and negative control (NC), with 3 replicates in each group

In this study, a follow-up investigation was conducted to examine the mechanism through which butyrate influences PEDV infection. RNA-seq was employed to analyze the transcriptional alterations in IPEC-J2 cells following PEDV infection after pre-treatment with butyrate. The PCA results demonstrated a clear distinction among the individual treatment groups (Fig. 2A). Additionally, the heatmap analysis revealed a notable up-regulation tendency in most of the genes following butyrate treatment, while the impact of PEDV on the host genes was comparatively less pronounced than that of butyrate treatment (Fig. 2B).

### Gene transcription alterations in IPEC-J2 cells after PEDV CV777 strain infection

**Fig. 3:**
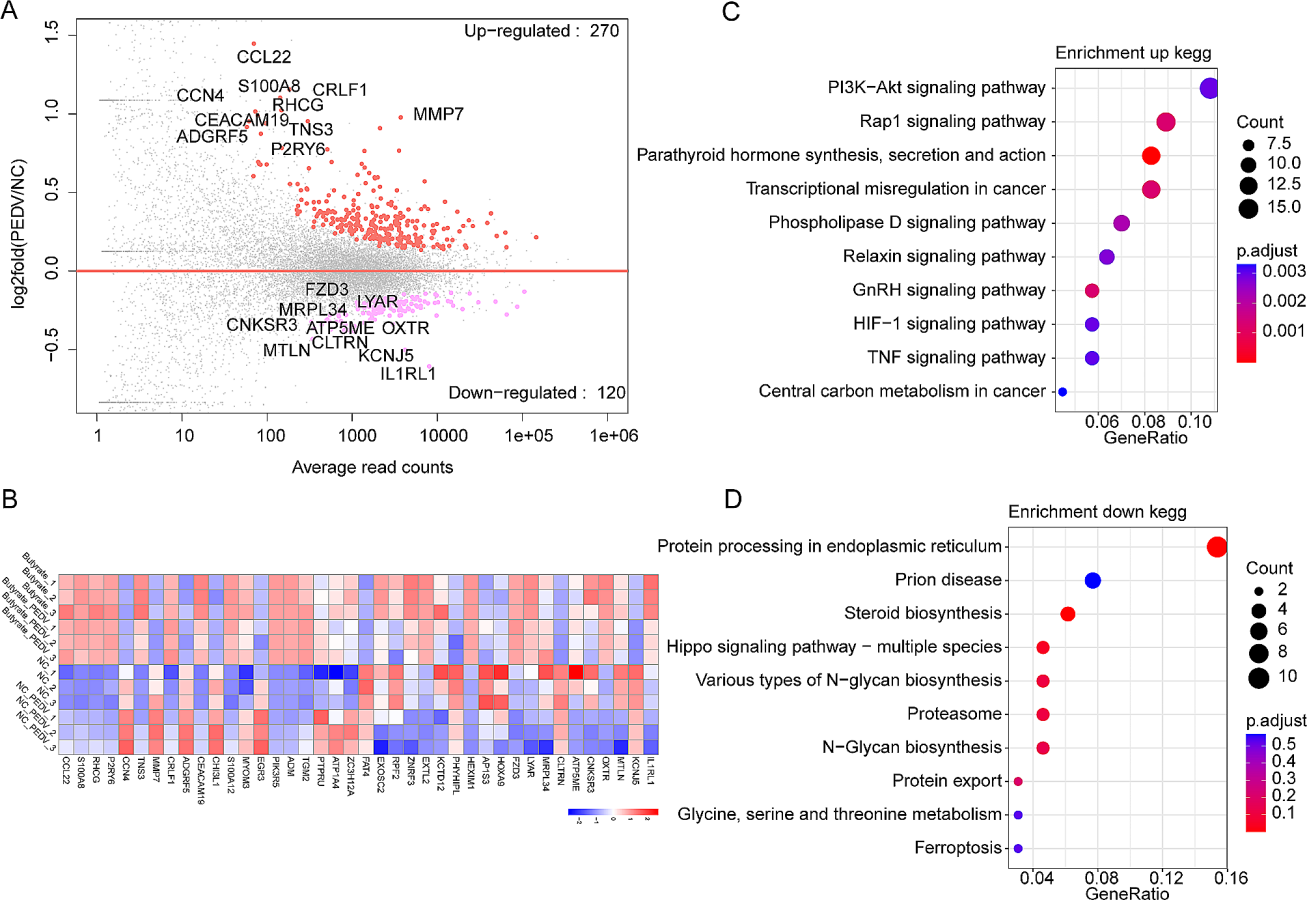
Illustrates the alterations in gene transcription observed in IPEC-J2 cells following infection with PEDV. (**A**) M-versus-A plot (MA) of DEGs between the PEDV-infected group and the NC group. Red dots represent up-regulated differentially expressed genes, pink dots represent down-regulated differentially expressed genes, and gray dots represent genes that are not significantly different. Horizontal coordinates represent average read counts, while vertical coordinates represent log2-transformed multiplicity of differences. (**B**) Heatmap of top 20 up-regulated and down-regulated DEGs, respectively. Bar represents the gene expression level after homogenization treatment. (**C**) KEGG enrichment analysis of up-regulated DEGs between the PEDV-infected group and the NC group. (**D**) KEGG enrichment analysis of down-regulated DEGs between the PEDV-infected group and the NC group

The DEGs between the PEDV-infected group and the control group were analyzed. A threshold of *P*-adjust value < 0.05 was set, and it was found that a total of 270 up-regulated DEGs and 120 down-regulated DEGs were detected after PEDV infection (Fig. 3A). Cluster analysis revealed distinct expression patterns of the expression of these DEGs in the butyrate-treated group (Fig. 3B). Subsequent enrichment analysis of these DEGs demonstrated that the up-regulated DEGs were primarily enriched in signaling pathways such as PI3K-AKT and Rap signaling pathways (Fig. 3C). Conversely, the down-regulated DEGs were predominantly enriched in signaling pathways related to protein processing in the endoplasmic reticulum and prion disease (Fig. 3D).

### The impact of butyrate treatment on gene transcription in IPEC-J2 cells

**Fig. 4:**
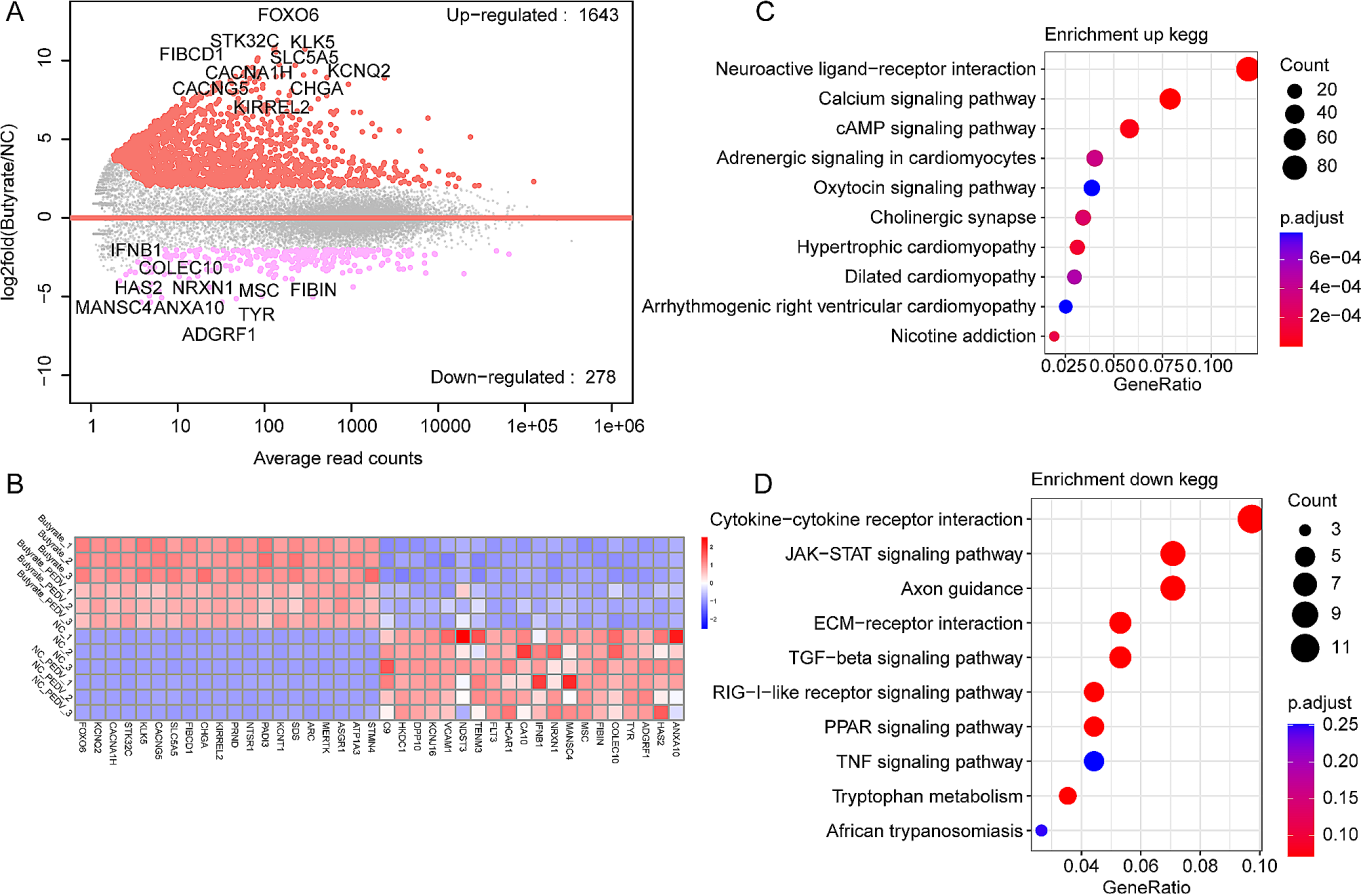
Effect of butyrate treatment on gene transcription in IPEC-J2 cells. (**A**) M-versus-A plot (MA) of DEGs between the butyrate group and the NC group. (**B**) Heatmap of top 20 up-regulated and down-regulated DEGs, respectively. (**C**) KEGG enrichment analysis of up-regulated DEGs between the butyrate group and NC the group. (**D**) KEGG enrichment analysis of down-regulated DEGs between the butyrate and the NC group

We conducted a further analysis of the DEGs between the group treated with butyrate and the control group. Given that butyrate treatment induced a broader spectrum of transcriptional alterations in host genes, we adjusted the threshold to *P*-adjust value < 0.05, |log2fold change| > 2. Consequently, we identified a total of 1,643 DEGs were up-regulated following butyrate treatment, while 278 DEGs were down-regulated (Fig. 4A). Cluster analysis revealed that these DEGs were primarily influenced by the administration of butyrate (Fig. 4B). Subsequent enrichment analysis of these DEGs demonstrated that the up-regulated DEGs were predominantly enriched in signaling pathways related to neuroactive ligand-receptor interaction and calcium signaling pathway (Fig. 4C). Conversely, the down-regulated DEGs were mainly enriched in pathways such as cytokine-cytokine receptor interaction, JAK-STAT signaling pathway, RIG-I-like receptor signaling pathway, and other pathways (Fig. 4D).

### The PI3K-AKT signaling pathway is an important potential regulatory pathway for inhibiting PEDV CV777 strain infection through butyrate treatment

**Fig. 5:**
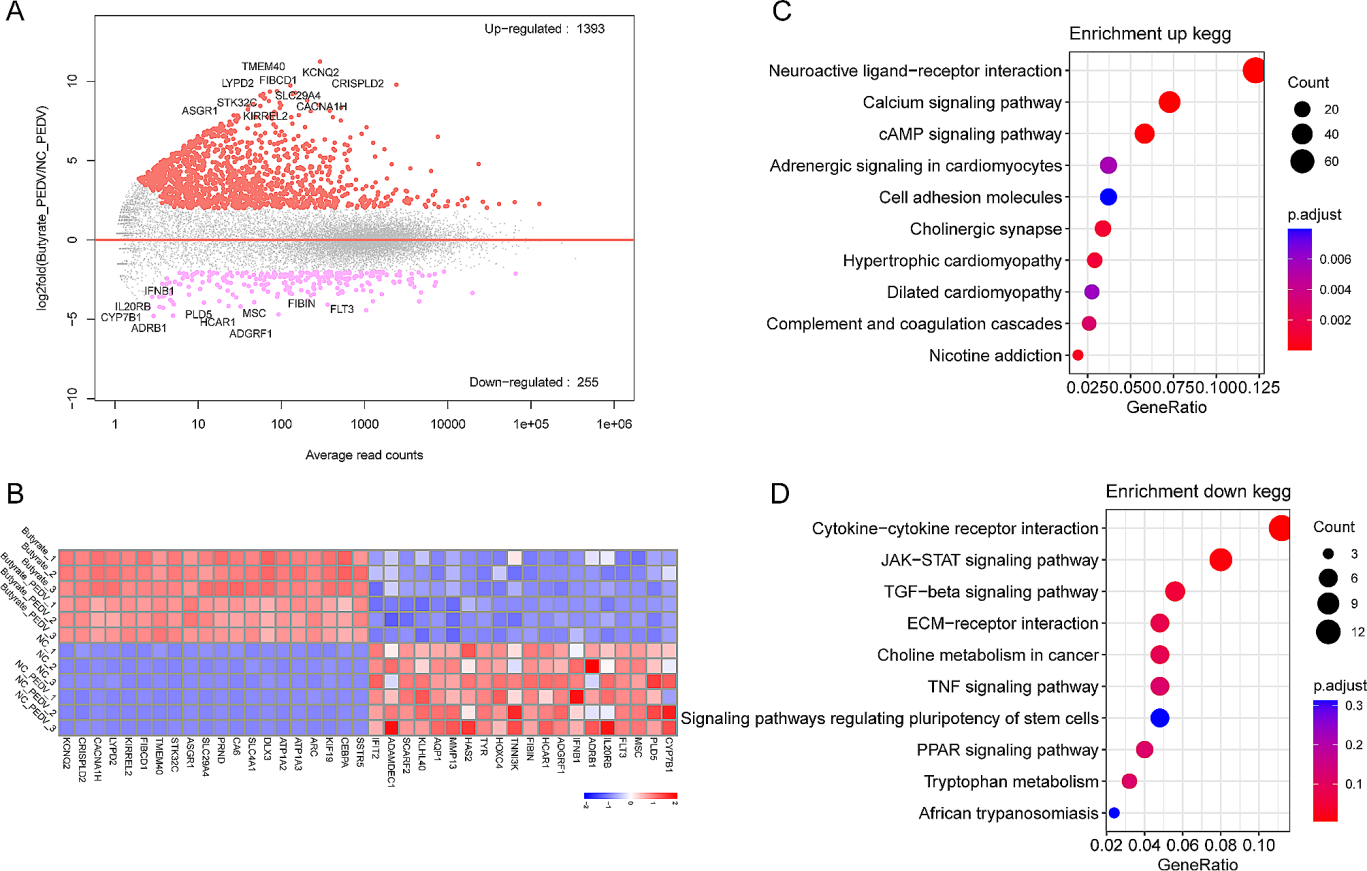
Effect of butyrate treatment on gene transcription to inhibit PEDV infection in IPEC-J2 cells. (**A**) M-versus-A plot (MA) of DEGs between the the group pretreated with butyrate and infected with PEDV, and the group infected with PEDV without pretreatment. (**B**) Heatmap of top 20 up-regulated and down-regulated DEGs, respectively. (**C**) KEGG enrichment analysis of up-regulated DEGs between the the group pretreated with butyrate and infected with PEDV, and the group infected with PEDV without pretreatment. (**D**) KEGG enrichment analysis of down-regulated DEGs between the group pretreated with butyrate and infected with PEDV, and the group infected with PEDV without pretreatment

**Fig. 6:**
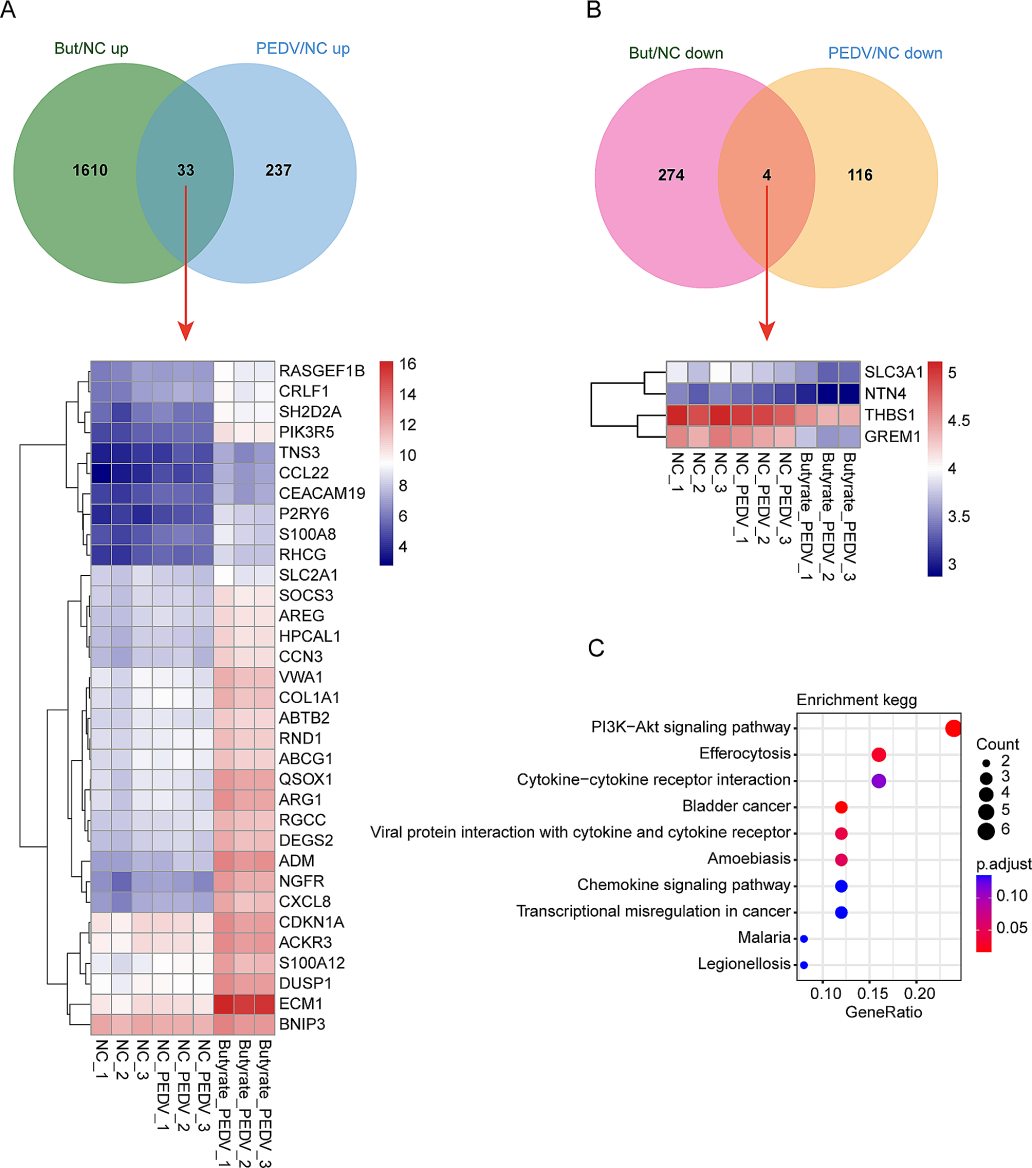
Overlap analysis for screening candidate regulatory genes. (**A**) Overlapped up-regulated genes in both the butyrate-treated and the PEDV-infected groups compared with the NC group. (**B**) Overlapped down-regulated genes in both the butyrate-treated and the PEDV-infected groups compared with the NC group. Heatmap below the Venn diagram represents the overlapped genes expression in different groups. (**C**) KEGG enrichment analysis of overlapped genes

In this study, the aim was to investigate the potential mechanism by which butyrate inhibits PEDV infection in IPEC-J2 cells. To achieve this, the DEGs were analyzed between the group pretreated with butyrate and infected with PEDV, and the group infected with PEDV without pretreatment. The thresholds of *P*-adjust value < 0.05 and |log2fold change| > 2 were set. It was found that a total of 1,393 up-regulated DEGs and 255 down-regulated DEGs were detected (Fig. 5A). Cluster analysis revealed that these DEGs are also mainly influenced by the administration of butyrate (Fig. 5B). Subsequent enrichment analysis of these up-regulated DEGs demonstrated that they were predominantly enriched in pathways such as neuroactive ligand-receptor interaction, calcium signaling pathway, cAMP signaling pathway, and other pathways, etc. (Fig. 5C). Conversely, the down-regulated DEGs were mainly enriched in pathways such as Cytokine-cytokine receptor interaction, JAK-STAT signaling pathway, TGF-beta signaling pathway (Fig. 5D). These pathways may play a potential role in the inhibition of PEDV infection by butyrate. Further, by overlapping the up-regulated DEGs after butyrate treatment and the up-regulated DEGs after PEDV infection, a total of 33 genes were identified. The heatmap analysis revealed that these up-regulated DEGs exhibited a higher expression trend (Fig. 6A). On the other hand, the down-regulated DEGs after butyrate treatment and PEDV infection were also overlapped, resulting in the identification of 4 genes that showed a lower expression trend after butyrate treatment (Fig. 6B). Furthermore, KEGG enrichment analysis of these overlapped genes indicated that they were primarily enriched in the PI3K-AKT signaling pathway (Fig. 6C), which was also up-regulated after PEDV infection (Fig. 3C). These findings suggest that butyrate may inhibit PEDV infection through modulation of the PI3K-AKT signaling pathway.

## Discussion

Butyrate, a carbon short-chain fatty acid synthesized by intestinal microorganisms through the metabolism of fiber, is present in the colon at concentrations reaching up to 140 mM [15], while in the jejunum, the concentration of butyric acid is around 5 mM [16]. It has been widely recognized for its potential to confer health benefits to humans, primarily through its anti-inflammatory properties. However, it has been suggested that butyrate may facilitate the replication of herpesviruses [17] as well as several RNA viruses [18–20]. Butyrate demonstrated a protective effect against influenza virus pathology in mice, despite an observed increase in viral titer [21]. PEDV, being an RNA virus, has been documented in a recent study to exhibit inhibitory effects on the replication of PEDV at concentrations ranging from 10 µM to 1 mM of butyrate [12]. However, it is worth noting that the concentration of butyrate in the intestinal tract of swine may exceed this range. Therefore, we opted to utilize a higher concentration of 5 mM to treat IPEC-J2 cells and observed comparable outcomes to those reported in the aforementioned study. It is postulated that within a safe concentration, butyrate has the potential to effectively diminish the infectivity of PEDV on intestinal epithelial cells. This suggests that butyrate may play a protective role in preventing PEDV infection in the intestine.

Previous research has primarily focused on the activation of G protein-coupled receptors and downstream NF-κB by SCFAs in response to viral infection [21–23]. This line of investigation has revealed that Butyrate treatment enhances the expression of interferon (IFN) and downstream interferon-stimulated genes (ISGs) during PEDV infection [12]. However, these studies have not comprehensively examined the overall transcriptional changes in the host. Consequently, we conducted a transcriptional landscape of the impact of butyrate pre-treatment on the progression of PEDV infection in IPEC-J2 cells using RNA-seq. The investigation of transcriptional changes in the host following PEDV infection has been extensively explored in recent studies [24–29]. However, there is some variability in the findings of different studies, including differences in the identified DEGs and signaling pathways. These discrepancies may be attributed to variations in the virulent strains, cells/individuals, and other factors. However, these studies have also identified several significant signaling pathways, including the PI3K-AKT signaling pathway [28] and the Rap1 signaling pathway [25], among others. These signaling pathways were found to be enriched in the DEGs following PEDV infection in IPEC-J2 cells. This observation suggests that these pathways may have potential regulatory functions during PEDV infection. Subsequently, the DEGs were analyzed following butyrate treatment. It was observed that butyrate treatment induced a broader spectrum of transcriptional alterations in host genes. Even after raising the threshold, a significantly higher number of DEGs were identified compared to those identified in response to PEDV infection. Furthermore, a majority of the genes exhibited an up-regulation trend following butyrate treatment. In contrast, PEDV infection did not exert as pronounced an effect on host genes as butyrate treatment, possibly due to the extensive host evasion mechanism employed by PEDV [30]. Our analysis of the DEGs indicated that several signaling pathways, including the JAK-STAT signaling pathway, ECM-receptor signaling pathway, RIG-I-like receptor signaling pathway, and TNF signaling pathway, were down-regulated following treatment with butyrate. This finding suggests that butyrate treatment may have an inhibitory effect on immune-related signaling pathways. It has been reported that the administration of butyrate resulted in an increase in the expression of over 800 genes. However, it was observed that butyrate strongly inhibited the expression of 60% of ISGs, while up-regulating 3% of ISGs [31]. These findings are consistent with the results obtained in the present study, suggesting that this may represent a novel mechanism through which butyrate exerts its effects on virus-infected cells.

To further investigate the potential targets of butyrate affecting PEDV infection, the DEGs were analyzed between the group pretreated with butyrate and infected with PEDV, and the group infected with PEDV without pretreatment. We found that these DEGs were enriched by pathways such as cytokine receptor interaction, JAK-STAT signaling pathway, neuroactive ligand-receptor interaction, and calcium signaling pathway. We found that the pathways enriched were similar to those enriched in the differential genes between the butyrate-treated and control groups. We hypothesized that this similarity might be attributed to the broader transcriptional changes induced by butyrate treatment. It is still debatable whether these pathways are associated with the inhibition of PEDV infection. Therefore, to further explore the differentially expressed genes and signaling pathways associated with the inhibition of PEDV infection by butyric acid, we analyzed the intersection of DEGs across various groups. The analysis revealed 37 genes that were found to be overlapped, and these genes were primarily enriched in the PI3K-AKT signaling pathway. It is worth noting that the PI3K/Akt signaling pathway has been reported to be up-regulated after PEDV infection and can inhibit PEDV replication [32]. However, it has also been reported that the PEDV nps6 protein of porcine epidemic diarrhea virus induces autophagy to promote viral replication through the PI3K/Akt/mTOR axis [33]. While there may be some discrepancies between the findings of these two studies, the military study demonstrated the significant involvement of the PI3K-AKT signaling pathway in PEDV infection. Butyrate might enhance tight junction protein abundance through mechanisms that include activation of AKT/mTOR-mediated protein synthesis [34]. This may be one of the reasons why high concentrations of butyrate can resist PEDV infection. This suggests that the PI3K-AKT signaling pathway may be one of the key pathways involved in the inhibition of PEDV replication by butyrate. Of course, there may be additional pathways and genes that are potentially significant targets for butyrate in regulating PEDV infection. However, further experimental evidence is required to substantiate these claims.

## Conclusion

This study presents evidence that Short-chain fatty acids inhibit PEDV CV777 strain replication, with butyrate being one of the most effective. butyrate has the potential to confer protection against PEDV CV777 strain infection in intestinal epithelial cells. Additionally, it sheds light on the host transcriptional profile of PEDV CV777 strain infection, which is found to be suppressed by butyrate. Our research indicates that the PI3K-AKT signaling pathway may play a potential role in mediating the regulatory effects of butyrate. This pathway could potentially serve as a novel strategy to inhibit PEDV infection through the actions of butyrate.

## Data Availability

No datasets were generated or analysed during the current study.

## Abbreviations

PEDV: Porcine epidemic diarrhea virus
PED: Porcine epidemic diarrhea
SCFAs: Short-chain fatty acids
IPEC-J2: Porcine small intestinal epithelial cells
MOI: Multiplicity of infection
Hpi: Hours post-infection
cDNA: Complementary DNA
RT-qPCR: Real-time quantitative PCR
PCA: Principal component analysis
DEGs: Differentially expressed genes
KEGG: Kyoto Encyclopedia of Genes and Genomes
IFN: Interferon
ISGs: Interferon-stimulated genes
